# Hydrothermal Synthesis of β-NiS Nanoparticles and Their Applications in High-Performance Hybrid Supercapacitors

**DOI:** 10.3390/nano14151299

**Published:** 2024-08-01

**Authors:** Xiaohong Liu, Yulin Wang, Chunwang Luo, Zheyu Zhang, Hongyan Sun, Chunju Xu, Huiyu Chen

**Affiliations:** 1 School of Energy and Power Engineering, North University of China, Taiyuan 030051, China; 2 School of Materials Science and Engineering, North University of China, Taiyuan 030051, China

**Keywords:** supercapacitors, β-NiS, hydrothermal synthesis, electrode materials, electrochemical energy storage

## Abstract

In this work, β-NiS nanoparticles (NPs) were efficiently prepared by a straightforward hydrothermal process. The difference in morphology between these NiS NPs was produced by adding different amounts of thiourea, and the corresponding products were denoted as NiS-15 and NiS-5. Through electrochemical tests, the specific capacity (*C_s_*) of NiS-15 was determined to be 638.34 C g^−1^ at 1 A g^−1^, compared to 558.17 C g^−1^ for NiS-5. To explore the practical application potential of such β-NiS NPs in supercapacitors, a hybrid supercapacitor (HSC) device was assembled with activated carbon (AC) as an anode. Benefitting from the high capacity of the NiS cathode and the large voltage window of the device, the NiS-15//AC HSC showed a high energy density (*E_d_*) of 43.57 W h kg^−1^ at 936.92 W kg^−1^, and the NiS-5//AC HSC provided an inferior *E_d_* of 37.89 W h kg^−1^ at 954.79 W kg^−1^. Both HSCs showed excellent cycling performance over 6000 cycles at 10 A g^−1^. The experimental findings suggest that both NiS-15 and NiS-5 in this study can serve as potential cathodes for high-performance supercapacitors. This current synthesis method is simple and can be extended to the preparation of other transition metal sulfide (TMS)-based electrode materials with exceptional electrochemical properties.

## 1. Introduction

As the economy continues to develop rapidly, traditional fossil fuels are being constantly consumed and environmental problems are becoming increasingly serious. These problems have accelerated the search for renewable sources such as solar, wind, and tidal energies, which can replace fossil fuels and some other traditional energy resources [1,2]. However, it is important to note that some renewable sources are susceptible to external factors, including geography and the environment, which are not conducive to the direct use of energy. Therefore, effective energy storage and conversion devices are required to mitigate energy instability and improve energy utilization [3]. Batteries and supercapacitors are regarded as common electrochemical energy storage systems today. Batteries can provide a high energy density (*E_d_*) and their runtimes are usually between an hour and several tens of hours [4,5]. However, batteries may face volume changes during a long-term charge–discharge process, which can slow down their charge rate and reduce lifespan, while supercapacitors can offer superior power density (*P_d_*), rapid charge–discharge behavior, preferable cyclic durability, an extended lifespan, and relatively cheap production costs, which make them a promising energy storage system [6,7,8]. Supercapacitors are currently used in many fields such as new energy vehicles, consumer electronics, and medical equipment [9,10].

The structure of a supercapacitor usually includes the electrode material, current collector, separator, and electrolyte. The overall performance of a supercapacitor may be affected by the properties of its electrode material to a large extent. Supercapacitors are typically classified as electric double-layer capacitors (EDLCs), pseudo-capacitors (PCs), or hybrid supercapacitors (HSCs), according to their electrode materials and energy storage mechanisms [11]. EDLCs are physical energy-storing devices. In the charge and discharge processes, the active material on the electrode surface undergoes continuous physical adsorption and desorption without involving any electrochemical reactions, thereby achieving efficient energy storage. Compared to conventional capacitors, EDLCs hold the advantages of enhanced energy storage capacitance, extended cycle life, and broader operating temperature range. Carbon-based materials stand as the most prevalent electrode materials for EDLCs [12]. PCs can realize charge storage through Faradaic reactions, wherein the electrode material processes a rapid and reversible redox reaction to enable energy storage and conversion. PCs can therefore provide a higher *E_d_* in addition to a larger *C_s_*. Owing to different energy storage principles, PCs can be grouped as redox, underpotential deposition, or intercalation PCs [13,14]. HSCs are usually assembled with a battery-type electrode material as the cathode and an EDLC electrode material as the anode. Typically, a cathode based on a battery-type electrode possesses high *C_s_*, and this type of cathode usually includes hydroxides, transition metal oxides (TMOs), and transition metal sulfides (TMSs). As for anodes utilizing carbon-based materials, they often exhibit good rate capability, a large potential window, and high *P_d_*. Consequently, HSCs can integrate some advantageous features of the two electrodes, and are expected to show promising application potential and development prospects [15,16].

In recent years, scientists have tried to develop novel electrode materials for supercapacitors in order to partially replace batteries in some areas that may cause harm to the environment. TMSs have received increasing attention because of their good redox performance, high theoretical specific capacity, and low preparation cost [17,18]. Moreover, TMSs usually exhibit better structural stability compared to that of TMOs and result in quick electron transport, which is probably due to the fact that sulfur is less electronegative than oxygen. At present, the most commonly used TMSs include MoS_2_, CuS, MnS, and NiS_2_ [19,20]. Nickel sulfide, NiS, possesses a simple chemical composition but a very remarkable theoretical *C_s_* of 1060 C g^−1^. Ni^2+^ shows good redox activity in alkaline electrolytes, and nickel-based electrode materials have been widely used in supercapacitors [21,22,23]. It is likely that NiS has attracted wide attention and achieved great progress in the field of supercapacitors because of its outstanding physical properties. The microstructures of NiS materials, including their shape, size, and surface properties, strongly determine their electrochemical performance. Currently, NiS powdered materials with different microstructures have been synthesized and applied in supercapacitors [24,25]. However, the shape and size of these powdered NiS materials are not easily controlled, and they usually suffer from structural instability and low electrical conductivity, which may influence further applications. For the sake of improving the electrochemical behaviors of powdered NiS materials, several methods, including the formation of binder-free structures, preparation of corresponding composites, metal doping, nanostructuring, morphology control, defect engineering, and so on, can be employed, and some of these methods have been successfully used for the preparation of other types of electrode materials [26,27,28].

Directly growing the active material onto a current collector can avoid the usage of binders and conductive reagents, which may reduce the internal resistance of an electrode and then improve its electrochemical property. For example, Edison et al. synthesized NiS nanosheets on nickel foam (NF) by the electrodeposition method in a mixed solution of nickel chloride and thiourea. This binder-free type electrode possessed a *C_s_* of up to 532 C g^−1^ at 2 A g^−1^ and held 94.9% of its initial capacity over 5000 cycles [29]. Additionally, combining NiS with one or more other electrode materials may resolve some problems existing in the practical usage of powdered NiS material. Meng et al. prepared Co_3_O_4_@NiS electrode material through a two-stage hydrothermal process. This composite delivered a remarkable *C_s_* as high as 697.65 C g^−1^ at 1 A g^−1^ and could retain 89.9% of its initial value over 5000 cycles at 10 A g^−1^ [30]. Although binder-free electrodes and composite electrode materials can exhibit superior electrochemical properties, they still face some negative issues in practical applications. Binder-free electrodes usually suffer from the poor adhesion of the active material on the current collector. Over a long period of the cyclic charge–discharge process, the active material is easily detached from the substrate, lowing the lifespan and cyclic stability of the electrode [31]. As for the composite electrode material, it often requires a relatively long time and tedious stages to prepare such powdered composites. Considering the cost and time consumption, the preparation of bare NiS powdered materials with excellent electrochemical performance is very important, and the controllable synthesis of NiS powdered materials with a uniform structure and morphology as well as good electrochemical properties is still challenging.

In this work, a straightforward hydrothermal approach was employed to prepare β-NiS NPs, during which different amounts of thiourea were used. These β-NiS NPs exhibited a high capacity and moderate rate performance. The HSC was assembled with such β-NiS NPs as the cathode and activated carbon (AC) as the anode, and the HSC showed good cycling stability over 6000 cycles and a high energy density of up to 43.57 W h kg^−1^ at 936.92 W kg^−1^. The current synthesis is simple and efficient, and can be extended for the preparation of other TMS-based electrode materials with superior properties in electrochemical energy storage and conversion.

## 2. Experimental Section

In a typical synthesis, 3 mmol of Ni(CH_3_COO)_2_·4H_2_O, 15 mmol of CS(NH_2_)_2_, and 10 mmol of CO(NH_2_)_2_ were dissolved in 40 mL of water with magnetic stirring, then the mixture was sealed in an autoclave and reacted for 12 h at 150 °C. The product was harvested, washed with water and ethanol, and dried at 70 °C. The sample was denoted as NiS-15. Other experiments were performed using similar procedures, except that 5, 10, and 30 mmol of CS(NH_2_)_2_ were, respectively, used, and the final samples were expressed as NiS-5, NiS-10, and NiS-30 accordingly. Detailed information regarding the structural characterizations and electrochemical tests is provided in the Supporting Information (S1 and S2).

## 3. Results and Discussion

**Figure 1:**
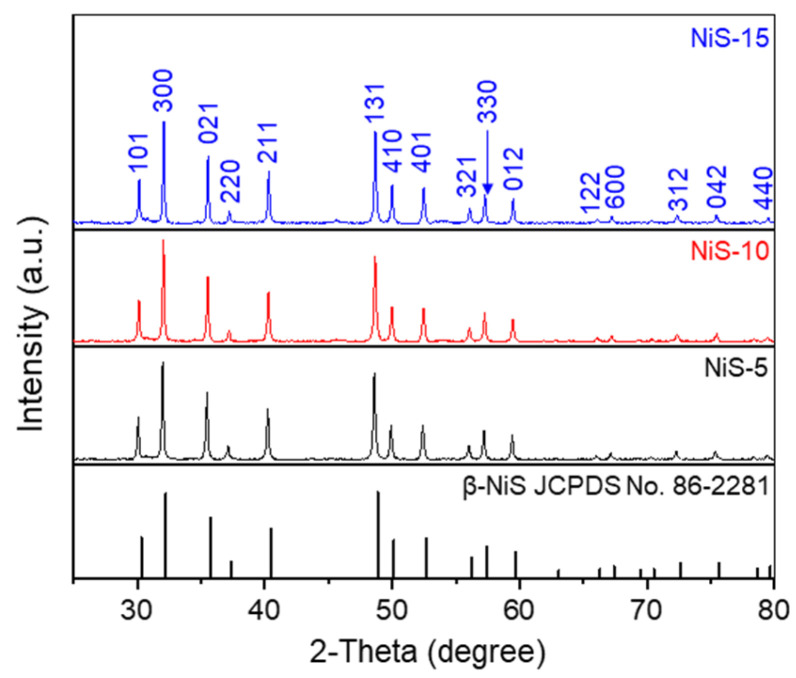
The XRD patterns of NiS-5, NiS-10, and NiS-15 electrode materials.

During the synthetic process, different amounts of thiourea were employed, and the products prepared with the amounts of 5, 10, 15, and 30 mmol thiourea were denoted as NiS-5, NiS-10, NiS-15, and NiS-30, respectively. Figure 1 shows the XRD patterns of NiS-5, NiS-10, and NiS-15. For the first sample, its diffraction peaks at 2θ values of 30.06°, 32.00°, 35.48°, 37.14°, 40.24°, 48.60°, 49.90°, 52.38°, 56.02°, 57.20°, 59.42°, 65.98°, 67.16°, 72.30°, 75.36°, and 79.44° correspond to the (101), (300), (021), (220), (211), (131), (410), (401), (321), (330), (012), (122), (600), (312), (042), and (440) crystal planes of β-NiS (JCPDS No. 86-2281) [32]. No other peaks regarding impurities were observed, indicating that the obtained β-NiS materials were highly pure. Figure S1a shows the XRD pattern of NiS-30; there are three additional peaks at 34.66°, 45.94°, and 53.46°, corresponding to the (101), (102), and (110) planes of α-NiS (JCPDS No. 75-0613). It is suggested that the crystal structure of NiS could be influenced by increasing the amount of thiourea during the synthetic procedure. When the dosage of thiourea was increased to 30 mmol, the crystal phase of NiS changed from R3m (160) to a mixed crystal phase of R3m (160) and P63/mmc (194) [33]. The percentages of β-NiS and α-NiS in the NiS-30 sample were determined to be 91.0 wt% and 9.0 wt%, respectively, and these values were calculated with the assistance of Jade 6 software.

**Figure 2:**
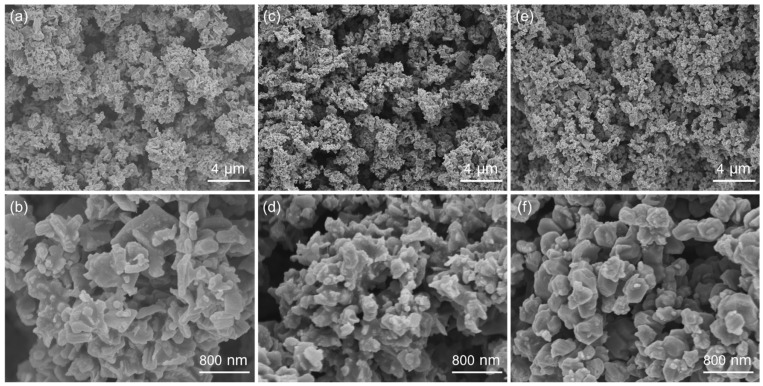
SEM images of NiS electrode materials prepared with different amounts of thiourea: (**a**,**b**) 5 mmol, (**c**,**d**) 10 mmol, and (**e**,**f**) 15 mmol.

**Figure 3:**
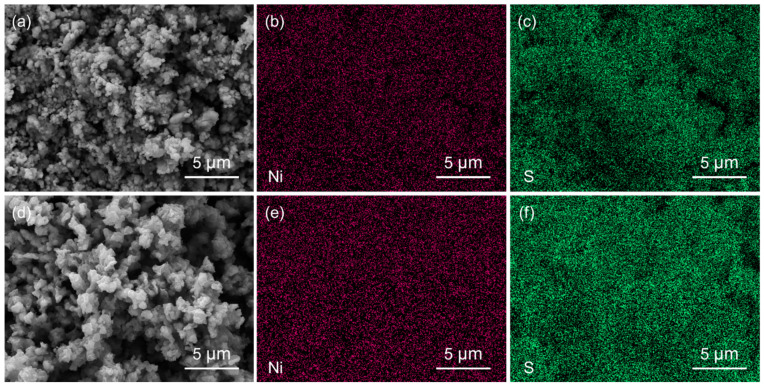
(**a**) SEM images of NiS-15 with the corresponding EDS element mappings of (**b**) Ni and (**c**) S, and (**d**) SEM images of NiS-5 with the EDS element mappings of (**e**) Ni and (**f**) S.

The shape and size of these NiS materials were investigated by SEM. As shown in Figure 2a, the NiS-5 sample contained a lot of nanoparticles (NPs). From the magnified SEM image in Figure 2b, it can be seen that a few large nanosheet-like microstructures with side lengths of about 0.5–1 μm coexist with these NPs. Figure 2c,d present the SEM images of NiS-10, showing that the NPs with irregular size tended to aggregate, and no nanosheet structure was found. When 15 mmol of thiourea was employed, the final particles were increased to 200–500 nm in size (Figure 2e,f). As for the sample prepared with 30 mmol of thiourea, the NiS particles became smooth in the surface and the size was decreased to 120–240 nm (Figure S1b,c). Considering the phase purity and microstructure of NiS nanomaterials, NiS-15 and NiS-5 were chosen for further structural characterizations and electrochemical tests. Figure 3 illustrates the SEM images of NiS-15 and NiS-5 along with their corresponding elemental mapping images, and both Ni and S elements were evenly distributed in these NiS nanomaterials.

**Figure 4:**
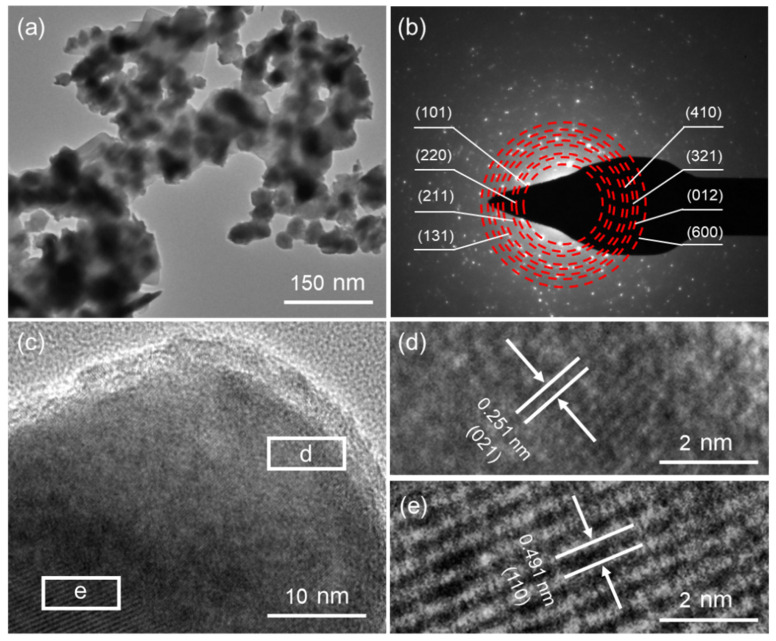
(**a**) TEM image, (**b**) SAED pattern, and (**c**–**e**) HRTEM images of NiS-15.

The TEM technique was employed to investigate the microstructures of NiS, and Figure 4a presents the typical TEM image of NiS-15, demonstrating that many NiS NPs were aggregated together. The SAED pattern is shown in Figure 4b, from which relatively clear ED rings can be seen, indicating the good polycrystalline property of NiS-15 NPs. The HRTEM image in Figure 4c exhibits several domains with different lattice orientations. In the magnified HRTEM images (Figure 4d,e), the interplanar distances were calculated to be 0.251 and 0.491 nm, corresponding to the (021) and (110) crystal planes of β-NiS, respectively. Figure S2a shows the TEM image of NiS-5, from which the presence of nanosheets was observed. The SAED pattern of the NiS-5 nanosheets is shown in Figure S2b, in which the ED spots are difficult to clearly index. Regular lattice spacings can be observed in the HRTEM image (Figure S2c). The interplanar distance in the magnified image in Figure S2d was determined to be 0.254 nm, matching the (021) crystal plane of the NiS material. Although the lattice spacing showed a slight variation in these HRTEM images by comparing the standard values in the JCPDS card (No. 86-2281) with an interplanar spacing of 0.25124 nm for the (021) plane and 0.48095 nm for the (110) plane, the crystal planes in the HRTEM images can be clearly and accurately defined.

**Figure 5:**
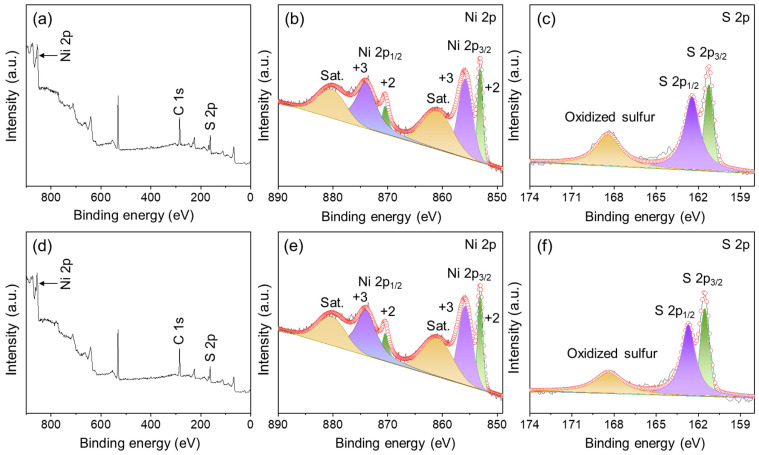
(**a**) The XPS survey spectrum of NiS-15, and the corresponding high-resolution spectra of (**b**) Ni 2p and (**c**) S 2p, (**d**) the XPS survey spectrum of NiS-5, and the corresponding core-level spectra of (**e**) Ni 2p and (**f**) S 2p.

The composition and chemical states of NiS-5 and NiS-15 were determined by using XPS characterization, and the results are shown in Figure 5. C1s at the binding energy of 284.6 eV was used as a standard for calibration. Figure 5a–c illustrate the XPS spectra of NiS-15, and Ni 2p, C 1s, and S 2p coexisted within the survey spectrum (Figure 5a). It can be observed in Figure 5b that there are two noticeable peaks at 855.7 and 873.6 eV, indicating the existence of Ni^3+^. Additionally, two other peaks were found at 852.9 and 870.3 eV, which were identified as Ni^2+^. The peaks at 860.6 and 880.0 eV corresponded to the shake-up satellite peaks [29,34]. Even though Ni^3+^ was detected, no other notable peaks associated with the nickel oxides could be found in the corresponding XRD pattern. This indicates that the occurrence of Ni^3+^ is ascribed to the surface oxidation of NiS material, and a similar phenomenon has been reported in earlier work [35,36]. The two main peaks and one satellite peak of S 2p are depicted in Figure 5c. The two signals at 161.2 and 162.4 eV belonged to S 2p_3/2_ and S 2p_1/2_, respectively. The former originated from the existence of S^2−^ and the latter was probably associated with the low coordination of S [37]. The peak at 168.4 eV belongs to oxidized sulfur, which is generated by the oxidization of sulfur outside of the material [38,39]. In addition, the XPS spectra of NiS-5 are illustrated in Figure 5d–f, and the composition as well as the chemical valence states are very similar to those of NiS-15.

**Figure 6:**
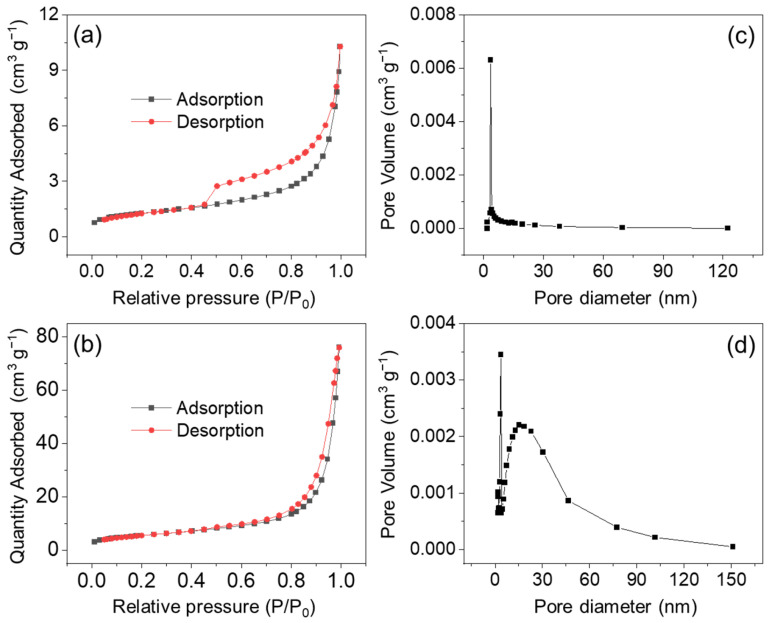
The N_2_ adsorption–desorption isotherms of (**a**) NiS-15 and (**b**) NiS-5, and the corresponding BJH pore size distribution of (**c**) NiS-15 and (**d**) NiS-5.

N_2_ adsorption–desorption tests can provide the porous information of NiS materials. As the curves presented in Figure 6a,b show, both curves exhibit type IV features with an H3 hysteresis loop, indicating the mesoporous structure of NiS-15 and NiS-5 [40]. The detailed porous data are presented in Table 1. Specifically, the specific surface area of NiS-15 and NiS-5 was calculated to be 4.46 and 20.25 m^2^ g^−1^, respectively. Figure 6c,d display the pore size distribution in the BJH model. The mean pore diameters were, respectively, determined as 10.1 and 19.7 nm. A porous structure with an appropriate pore size distribution is helpful for the charge transfer and ion diffusion of the electrode material, and so the electrochemical performance can be expected to be improved to a certain extent.

**Figure 7:**
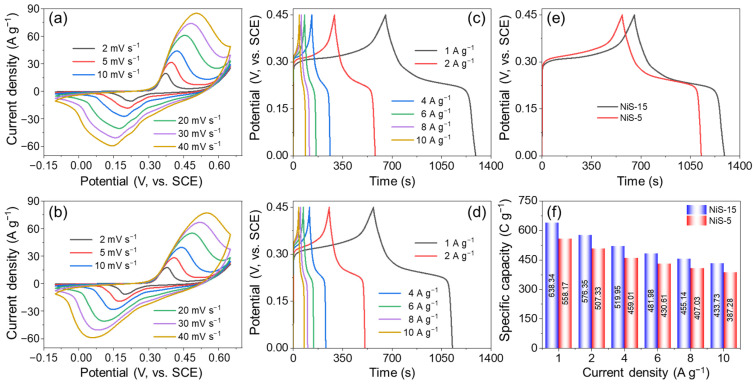
CV curves of (**a**) NiS-15 and (**b**) NiS-5 at various scan rates; GCD curves of (**c**) NiS-15 and (**d**) NiS-5 at various current densities; (**e**) GCD curves of NiS-15 and NiS-5 at 1 A g^−1^; and (**f**) rate performance of both NiS-15 and NiS-5.

The electrochemical properties of these NiS electrode materials were initially evaluated in a three-electrode setup. Figure 7a,b show the CV curves of NiS-15 and NiS-5 at a scan rate ranging from 2 to 40 mV s^−1^. Evident redox peaks were observed, indicating that the NiS was a battery-type electrode material. With the scan rate increasing, both the anodic and cathodic peaks shifted to extreme values. As the scan rate was high enough, only the area outside of the electrode participated in the Faradic reactions [41]. Additionally, all the CV curves remained in a similar shape without any obvious change, suggesting the good reversibility of the redox reactions. The *b* value can be calculated by *i* = *av^b^*, where *i* is the peak current (mA), *a* and *b* are constants, and *v* is the scan rate (mV s^−1^) [42,43]. The results are shown in Figure S3a,b. In theory, if *b* equals 0.5, the capacity is dominated by an ion-diffusion process. Otherwise, the surface capacitance plays a dominant role if *b* = 1. For the NiS-15 electrode, the *b* values for anodic and cathodic current were calculated to be 0.487 and 0.573, respectively, and for the NiS-5 electrode, the values were 0.490 and 0.543, although all the *b* values deviated a little from 0.5, demonstrating that ion diffusion was the main mechanism of energy storage for NiS electrode materials. The detailed capacity contribution can be determined by the equation of *i* = *k*_1_*v* + *k*_2_*v*^1/2^, where *k*_1_ and *k*_2_ are the constants influenced by the capacitive (surface-controlled) and diffusion-controlled processes, respectively [44,45], and the results are illustrated in Figure S4. At the scan rate of 10 mV s^−1^, the contribution of the surface capacity was calculated to be 67.4% for NiS-15 and 64.7% for NiS-5, respectively. With the scan boosted from 2 to 40 mV s^−1^, the capacity contribution of ion diffusion was decreased from 48.8% to 4.8% for NiS-15, while for the NiS-5 electrode, the value was decreased from 50.5% to 4.7%. Hence, to achieve the ion-diffusion-dominated capacity, the scan rate should be as low as enough.

Figure 7c,d show the GCD profiles performed over the potential window of 0–0.45 V. Both curves were nonlinear with obvious charge and discharge platforms, suggesting that the NiS was ascribed to a battery-type electrode material. In addition, these GCD curves were basically symmetrical at any current density, showing the good reversibility of Faradic reactions. At the current densities of 1, 2, 4, 6, 8, and 10 A g^−1^, the *C_s_* of NiS-15 were determined to be 638.34, 576.35, 519.95, 481.98, 455.14, and 433.73 C g^−1^, respectively. As for the NiS-5 electrode, the *C_s_* were 558.17, 507.33, 459.01, 430.61, 407.03, and 387.28 C g^−1^, accordingly. Figure 7e shows the GCD curves of NiS-15 and NiS-5 under the current load of 1 A g^−1^, and it was obvious that the NiS-5 electrode discharged for a shorter time than that of the NiS-15 electrode. Figure 7f presents the rate performance of NiS-15 and NiS-5, and the two electrodes exhibited similar rate capabilities of 67.9% and 69.4%, respectively. The highest capacity (638.34 C g^−1^) of NiS-15 is superior to some nickel sulfide powder materials reported previously, but this value is still lower than some nickel sulfide-based composites and binder-free electrode materials (Table 2).

**Figure 8:**
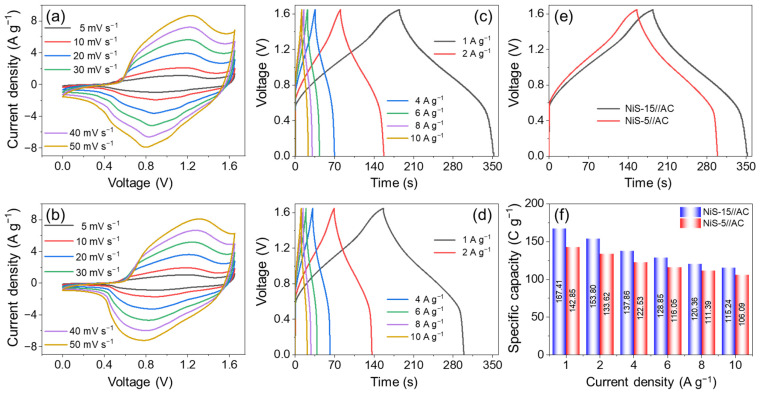
The CV curves of (**a**) NiS-15//AC HSC and (**b**) NiS-5//AC HSC at different scan rates, the GCD curves of (**c**) NiS-15//AC HSC and (**d**) NiS-5//AC HSC at different current densities, (**e**) the GCD curves of both HSCs at 1 A g^−1^, and (**f**) the rate performance of both HSCs.

To evaluate the practical application of these NiS electrode materials in the field of electrochemical energy storage, a hybrid supercapacitor (HSC) device was assembled with NiS-15 (NiS-5) as the cathode and activated carbon (AC) as the anode, and the devices were denoted as “NiS-15//AC HSC” and “NiS-5//AC HSC”, respectively. The AC material often exhibits good merits such as superior rate performance, broad potential window, and great power density. More importantly, it shows excellent chemical stability in the alkaline solution electrolyte. Hence, the AC is widely used as a negative electrode for the assembly of an HSC device. The active materials loaded on the cathode and anode should follow the charge balance to achieve the optimal electrochemical performance [60]. Figure S5 depicts the CV curves of both NiS-15 and AC materials in a three-electrode system. The AC was operated over a potential window of −1.0–0 V, while for the NiS-15, the potential window was −0.1–0.65 V. As a result, the voltage window for the electrochemical tests of NiS//AC HSC devices in the two-electrode system was determined from 0 to 1.65 V. The CV tests for HSCs were conducted at scan rates ranging from 5 to 50 mV s^−1^, and the results are presented in Figure 8a,b. All the CV profiles exhibited consistent irregular rectangles and displayed mild redox peaks, demonstrating that the *C_s_* of the HSC was generated from battery-type NiS and EDLC-based AC. As the scan was boosted, there was no obvious change in the CV shapes, suggesting the good reversibility of redox reactions [61]. In addition, the enclosed area in the CV curve of NiS-15//AC HSC was slightly larger than that of NiS-5//AC HSC at any scan rate, indicating the higher capacity that NiS-15//AC HSC could deliver. Figure 8c,d displays the GCD curves of HSCs at 1–10 A g^−1^. Each GCD curve showed a nonlinear shape with a weak charge and discharge plateau, further demonstrating that the battery-like NiS cathode and EDLC-type AC anode contributed to the total capacity of HSC. Figure 8e shows the GCD profiles of HSCs at the current load of 1 A g^−1^, and a longer discharge time of NiS-15//AC HSC was observed. The rate performance is compared in Figure 8f, and the *C_HSC_* of the device at each current density was calculated. The NiS-15//AC delivered *C_HSC_* values of 167.41, 153.80, 137.86, 128.85, 120.36, and 115.24 C g^−1^ at 1, 2, 4, 6, 8, and 10 A g^−1^, respectively. In comparison, the NiS-5//AC HSC showed 142.85, 133.62, 122.53, 116.05, 111.39, and 106.09 C g^−1^, accordingly. The NiS-15//AC HSC showed a 68.8% rate capability at 10 A g^−1^, slightly lower than 74.3% for NiS-5//AC HSC.

**Figure 9:**
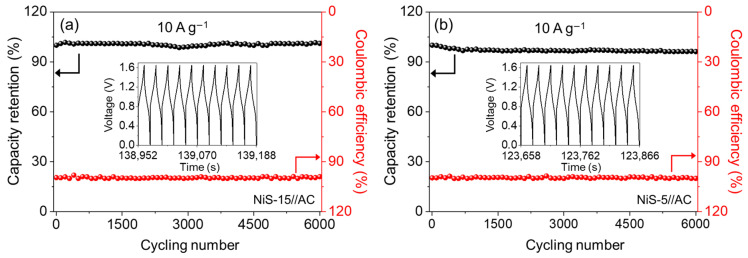
The cycling performance and Coulombic efficiency of (**a**) NiS-15//AC HSC and (**b**) NiS-5//AC HSC during 6000 cycles at 10 A g^−1^; the insets show the last 10 GCD curves.

The long-term cycling performance is an important issue when assess the practical application potential of HSCs. Herein, the cycling test was performed over 6000 GCD at a high current density of 10 A g^−1^. The NiS-15//AC HSC could keep 101.1% of its initial capacity at the end of the 6000th cycle (Figure 9a), while for NiS-5//AC HSC, about 96.2% of the original capacity was retained (Figure 9b). The insets present the last 10 cycles, which still maintained the initial shape of the GCD curve. Meanwhile, the Coulomb efficiency of both HSCs was close to 100% during the total cycling process, verifying the good reversibility of the Faradic reactions. The cycling property supported the point that the NiS electrode materials in this work exhibited excellent long-term cycling stability.

**Figure 10:**
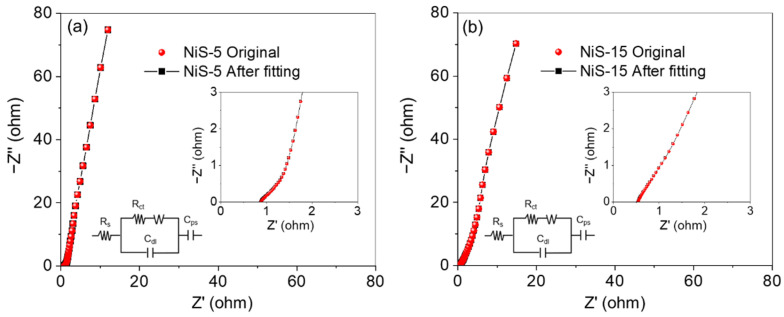
The Nyquist plots of (**a**) NiS-5 and (**b**) NiS-15 along with their plots fitted by ZSimDemo before cycling, and the enlarged Nyquist plots along with the corresponding simulated equivalent circuits in the insets.

The resistance of the NiS electrode materials was investigated by EIS tests over a frequency of 10^−2^–10^5^ Hz. Figure S6 presents the Bode plots, and Figure 10 shows the original and fitting Nyquist plots along with the corresponding equivalent circuit diagrams before cycling. Each plot contained a semicircle in the high-frequency region and a straight line in the low-frequency region. The intercept of the semicircle in the real axis corresponds to the internal resistance (*R_s_*), and the diameter of the semicircle represents the charge transfer resistance (*R_ct_*) [62,63]. The initial *R_s_* value was directly determined from the intercept in the real axis, and the values were 0.54 Ω for NiS-15 and 0.88 Ω for NiS-5. The *R_ct_* values were obtained after fitting with the ZSimDemo 3.30d software, and the values were 0.1 and 0.25 Ω, respectively. Both straight lines were steep, which indicated the low resistance of ion diffusion (Warburg impendence, *Z_w_*). Hence, both the NiS-15 and NiS-5 electrodes possessed good conductivity with low resistance. After 6000 cycles, the *R_s_* and *R_ct_* of NiS-15 were increased to 0.64 and 1 Ω, respectively, and for NiS-5, the values were 0.99 and 1.04 Ω, accordingly (Figure S7). Table S1 lists all the components used in the equivalent circuit diagram of the EIS model along with their fitting parameters, and it provided the standard deviation of the fitted statistics to evaluate its fitting quality.

**Figure 11:**
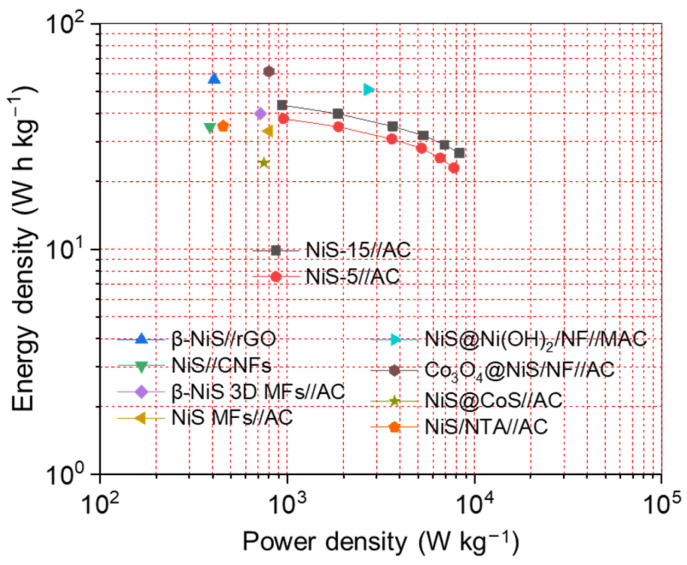
The Ragone plots of the NiS-15//AC HSC, NiS-5//AC HSC, and some other NiS-based HSCs reported earlier.

The *P_d_* and *E_d_* of HSC are also important factors in determining its commercialization potential, and the detailed values of *P_d_* and *E_d_* can be calculated from the GCD curves. The NiS-15//AC HSC delivered an *E_d_* of 43.57 W h kg^−1^ at the *P_d_* of 936.92 W kg^−1^, and could still hold 26.66 W h kg^−1^ as the *P_d_* was improved to 8328.15 W kg^−1^. As for the NiS-5//AC HSC, it could produce 37.89 W h kg^−1^ at the *P_d_* of 954.79 W kg^−1^. Figure 11 presents the Ragone plots of NiS-15//AC HSC, NiS-5//AC HSC, and some other NiS-based HSCs for a comparison. The highest *E_d_* of 43.57 W h kg^−1^ in this work was superior to those of NiS//carbon nanofibers (NiS//CNFs, 34.9 W h kg^−1^) [48], β-NiS 3D microflowers//AC (β-NiS 3D MFs//AC, 40 W h kg^−1^) [50], and NiS microflowers//AC (NiS MFs//AC, 33.4 W h kg^−1^) [37]. The value was also higher than those of some HSCs assembled with NiS-based composites such as NiS@CoS//AC (24.1 W h kg^−1^) [52] and NiS/Nitrilotriacetic Acid//AC (NiS/NTA//AC, 35.1 W h kg^−1^) [7]. However, the *E_d_* was still inferior to those of β-NiS//reduced graphene oxide (β-NiS//rGO, 56.6 W h kg^−1^) [38], NiS@Ni(OH)_2_/NF//modified activated carbon (NiS@Ni(OH)_2_/NF//MAC, 51 W h kg^−1^) [56], and Co_3_O_4_@NiS/NF//AC (61.34 W h kg^−1^) [30]. Considering the high *E_d_* and good cycling stability of NiS-15//AC HSC, NiS-15 may be promising as an electrode material in the field of electrochemical energy storage.

## 4. Conclusions

In summary, β-NiS powdered materials were successfully synthesized via a straightforward hydrothermal process, and their electrochemical performance was evaluated in this work. The main results can be summarized as follows: (1) The amount of thiourea in the synthesis played an important role in determining the size, shape, and crystal phase of the final product. In particular, some α-NiS emerged and coexisted when 30 mmol of thiourea was employed. (2) These NiS electrode materials exhibited a battery-type electrochemical response. In the three-electrode system, the NiS-15 delivered a specific capacity of 638.34 C g^−1^ at the current density of 1 A g^−1^, higher than the 558.17 C g^−1^ of the NiS-5 electrode, and both electrodes showed a similar rate performance (67.9% vs. 69.4%) at 10 A g^−1^. (3) The HSC device was assembled with NiS as the cathode and AC as the anode to evaluate the practical application potential of these NiS materials. The NiS-15//AC HSC displayed a superior *E_d_* of 43.57 W h kg^−1^ at 936.92 W kg^−1^. After 6000 GCD cycles at 10 A g^−1^, both devices presented a remarkable cycling stability with 101.1% and 96.2% capacity retention, respectively. (4) These results suggest that β-NiS can serve as an advanced cathode for the assembly of high-performance hybrid supercapacitors. The current synthesis method can be applied to the preparation of other TMS-based electrode materials with exceptional electrochemical properties along with their applications in electrochemical energy storage.

## Figures and Tables

**Table 1:** The porous data of the NiS electrode materials.

Materials	Specific Surface Area (m^2^ g^−1^)	Average Pore Size (nm)
NiS-5	20.25	19.7
NiS-15	4.46	10.1

**Table 2:** The specific capacities of some nickel sulfide-based electrode materials.

Materials	Methods	Electrolyte (KOH)	Potential Window (V)	Specific Capacity (C g^−1^)	Refs.
Flower-like NiS	Solvothermal	6 M	0–0.45	271.79 @ 1 A g^−1^ (603.97 F g^−1^)	[46]
α-NiS nanoparticles	Solvothermal	6 M	0–0.5	400 @ 0.5 A g^−1^ (800 F g^−1^)	[47]
NiS hierarchical hollow cubes	Anion exchange and calcination	2 M	0–0.55	480.98 @ 1 A g^−1^ (874.5 F g^−1^)	[48]
NiS microflowers	Sacrificial template	3 M	0–0.45	505.22 @ 1 A g^−1^ (1122.7 F g^−1^)	[49]
NiS microflowers	Sacrificial template	3 M	0–0.45	591.93 @ 1 A g^−1^ (1315.4 F g^−1^)	[37]
β-NiS 3D microflowers	Hydrothermal	1 M	0–0.45	688.05 @ 2 A g^−1^ (1529 F g^−1^)	[50]
NiS hollow spheres	Hydrothermal and annealing	2 M	0–0.7	753.2 @ 1 A g^−1^ (1076 F g^−1^)	[51]
Cabbage-like α-NiS	Solvothermal and annealing	6 M	0–0.45	849.17 @ 1 A g^−1^ (235.88 mA h g^−1^)	[25]
NiS@CoS	Hydrothermal and electrodeposited	2 M	0–0.5	605 @ 1 A g^−1^ (1210 F g^−1^)	[52]
NiS@Cu_7_S_4_	Solvothermal	6 M	0–0.5	837 @ 1 A g^−1^ (1674 F g^−1^)	[53]
Wrinkle-shaped NiS/NF	Solvothermal	2 M	−0.1–0.4	385 @ 1 A g^−1^ (770 F g^−1^)	[54]
NiS/NF	Solvothermal	3 M	0–0.45	1164.15 @ 0.2 A g^−1^ (2587 F g^−1^)	[55]
NiS@Ni(OH)_2_/NF	Ionic layer adsorption and reaction	3 M	0–0.65	108 @ 3 A g^−1^	[56]
NiS@CoO/NF	Electrodeposition and annealing	3 M	−0.05–0.45	527 @ 6 A g^−1^ (1054 F g^−1^)	[57]
Co_3_O_4_@NiS/NF	Hydrothermal, annealing, and hydrothermal	6 M	0–0.5	697.65 @ 1 A g^−1^ (1395.3 F g^−1^)	[30]
B-Ni_x_S_y_/C	Pyrolysis, hydrothermal vulcanization	3 M	0–0.43	1250.4 @ 1 A g^−1^	[58]
Flower-like Ni_3_S_2_/NiO	Hydrothermal, successive ionic layer adsorption reaction	1 M	0–0.7	1453.98@ 1 A g^−1^ (2077.12 F g^−1^)	[59]
NiS-15	Hydrothermal	2 M	0–0.45	638.34 @ 1 A g^−1^	This work

## Data Availability

Data is contained within the article or Supplementary Materials.

## Supplementary Materials

The following supporting information can be downloaded at: https://www.mdpi.com/article/10.3390/nano14151299/s1, Figure S1: (a) XRD pattern and (b,c) SEM images of NiS-30; Figure S2: (a) TEM image, (b) SAED pattern, and (c,d) HRTEM images of NiS-5; Figure S3: The plots of log (peak current) versus log (scan rate) for the anodic and cathodic peaks of (a) NiS-15 and (b) NiS-5; Figure S4: The contribution of surface capacitance (red) and diffusion process (blue) to the total charge storage at 10 mV s^−1^ for (a) NiS-15 and (b) NiS-5, and the contribution of surface capacitance (red) and diffusion process (blue) to the total charge storage at different scan rates for (c) NiS-15 and (d) NiS-5; Figure S5: The CV curves of AC and NiS-15 at the scan rate of 10 mV s^−1^; Figure S6: The Bode plots of NiS-5 and NiS-15 electrode materials; Figure S7: The Nyquist plots of (a) NiS-5 and (b) NiS-15 along with their fitting plots by ZSimDemo after cycling, and the enlarged Nyquist plots along with the corresponding simulated equivalent circuits in the insets; Table S1: The components of the EIS equivalent circuits and their statistical parameters of modeling. References [64,65,66] are cited in the supplementary materials.
