# Supplementary material for: Hydrothermal Synthesis of β-NiS Nanoparticles and Their Applications in High-Performance Hybrid Supercapacitors
Source: Nanomaterials (Basel). 2024 Aug 1;14(15):1299. doi: 10.3390/nano14151299 (PMC11314592; doi:10.3390/nano14151299)
Supplement: Supplementary file 1 [file nanomaterials-14-01299-s001.zip › nanomaterials-3114855-supplementary.pdf]

nanomaterials

Supplementary Materials

Hydrothermal Synthesis of β-NiS Nanoparticles and Their Applications in High-Performance Hybrid Supercapacitors

Xiaohong Liu [1], Yulin Wang [2], Chunwang Luo [2], Zheyu Zhang [2], Hongyan Sun [2], Chunju Xu [2,*] and Huiyu Chen [2,*]

[1] School of Energy and Power Engineering, North University of China, Taiyuan 030051, China
[2] School of Materials Science and Engineering, North University of China, Taiyuan 030051, China
* Correspondence: chunju@nuc.edu.cn (C. Xu); hychen@nuc.edu.cn (H. Chen)

S1. Structural characterizations

The crystal structure and phase purity of the NiS were characterized by X-ray diffraction (XRD, Bruker D8 Advance Diffractometer) using Cu Kα radiation (λ = 0.15406 nm). The morphology was observed by scanning electron microscopy (SEM, JEOL JSM7100F). The elemental distributions in the NiS were revealed by energy-dispersive X-ray spectroscopy (EDS) element mappings in the same SEM equipment. The morphology and microstructure were further studied by transmission electron microscopy (TEM), high resolution TEM (HRTEM), and selected-area electron diffraction (SAED) pattern, which were taken on a JEOL JEM2100F transmission electron microscope with an acceleration voltage of 200 kV. The composition and chemical valence states of NiS were investigated by X-ray photoelectron spectroscope (XPS, ESCALAB 250). The surface characteristic including specific surface area and pore size distribution of these NiS electrode materials were studied by N_2 adsorption-desorption tests at 77 K using an ASAP 2020 (Micromeritics, America) equipment. Besides, the specific surface area was calculated through Brunauer-Emmett-Teller (BET) method, and the pore size distribution was obtained by Barret-Joyner-Halenda (BJH) model.

S2. Electrochemical tests

The NiS powder was mixed with acetylene black and polyvinylidene fluoride (PVDF) in a mass ratio of 80:15:5, and then was dispersed in *N*-methyl-2-pyrrolidone (NMP) solvent. The obtained slurry was coated on cleaned nickel foam and processed vacuum-drying at 115 °C for 24 h. Then the nickel foam with loaded active material was pressed under a pressure of 10 MPa, and then it served as working electrode. All electrochemical tests were performed on a CHI 660E electrochemical workstation (Shanghai Chenhua Instrument Co. LTD, China) using three-electrode and two-electrode system, respectively. In the three-electrode configuration, a Pt foil served as the counter electrode and a saturated calomel electrode (SCE) served as the reference electrode. The cyclic voltammetry (CV) curves were obtained at scan rate ranging from 2 to 40 mV s^{-1}, and the galvanostatic charge-discharge (GCD) tests were performed at current density of 1−10 A g^{-1}. Before carrying out the tests in two-electrode system, a hybrid supercapacitor (HSC) was assembled with activated carbon (AC) as anode. A solution of 2 M KOH was employed as electrolyte for all electrochemical tests. In this study, the same AC mentioned in the previous work with capacitance of 178.5 F g^{-1} at 1 A g^{-1} was used [1].

The specific capacity (C_s, C g^{-1}) of the electrode material can be calculated according to the Formula (1) [2]:

$$C_s = \frac{I \cdot \Delta t}{m} \quad (1)$$

Where I (A) is the current density in the GCD test, Δt (s) is the discharge time, and m (g) corresponds to the mass of NiS electrode material supported on the working electrode.

To assemble the HSC, the charge balance should be maintained according to the Formula (2) [3]:

$$\frac{m_+}{m_-} = \frac{C_S^- \cdot \Delta V^-}{Q_S^+} \tag{2}$$

Among them, m_+ (g) and m_- (g) represent the mass loading of NiS and AC, respectively, C_s^- (F g^{-1}) is the specific capacitance of AC, ΔV^- (V) is the potential window of AC, and Q_s^+ (C g^{-1}) corresponds to the specific capacity of NiS.

The specific capacity (C_{HSC}, C g^{-1}), energy density (E_d, W h kg^{-1}), power density (P_d, W kg^{-1}), and Coulombic efficiency (η, %) of HSC device are calculated according to the Formulas (3−6) [4]:

$$C_{HSC} = \frac{I \cdot \Delta t}{M} \tag{3}$$

$$E_d = \frac{\int_{t_1}^{t_2} I \cdot V(t)dt}{3.6M} \tag{4}$$

$$P_d = \frac{3600E}{\Delta t} \tag{5}$$

$$\eta = \frac{t_d}{t_c} \times 100\% \tag{6}$$

Where, M (g) is the total mass loading of NiS and AC. t_d (s) and t_c (s) represent the discharge time and charge time of HSC, respectively.

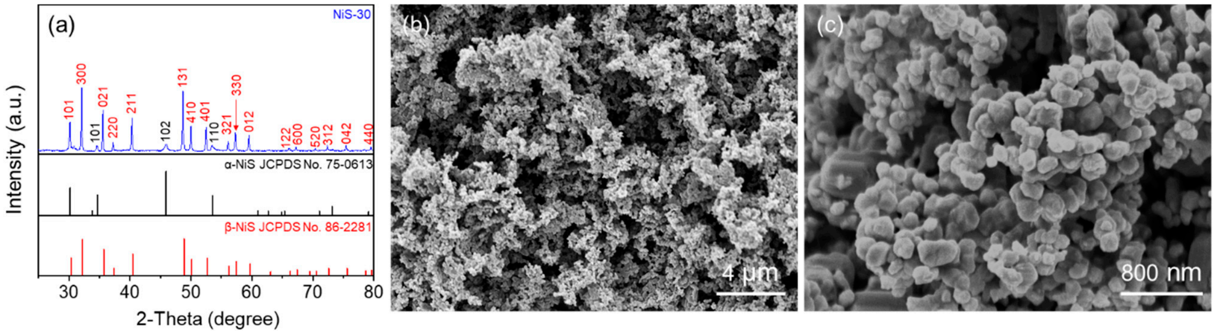

Figure S1. (a) XRD pattern and (b, c) SEM images of NiS-30.

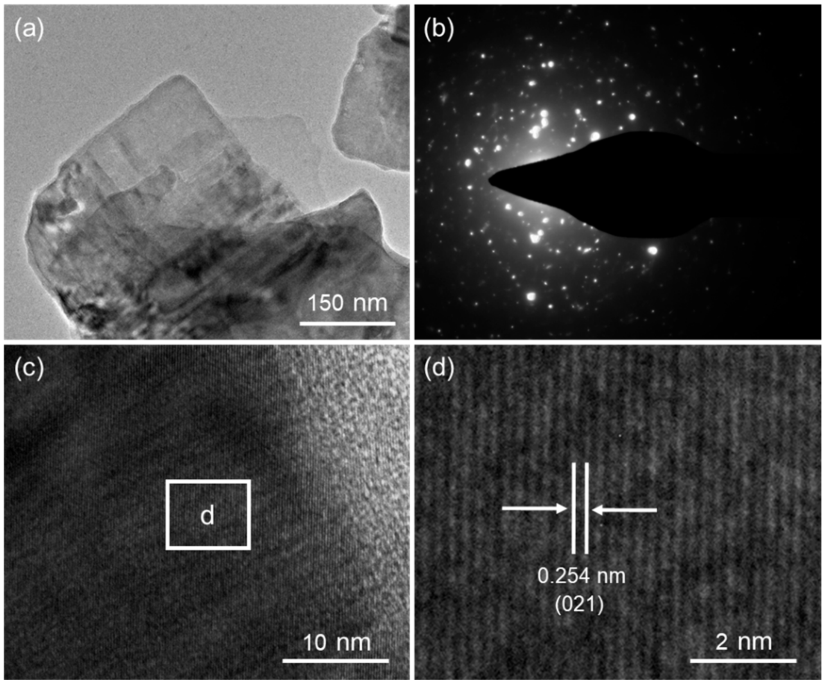

Figure S2. (a) TEM image, (b) SAED pattern, and (c, d) HRTEM images of NiS-5.

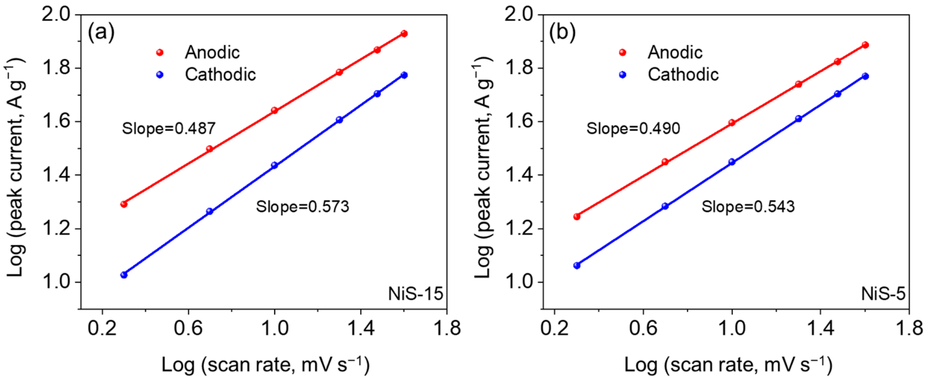

Figure S3. The plots of log (peak current) versus log (scan rate) for the anodic and cathodic peaks of (a) NiS-15 and (b) NiS-5.

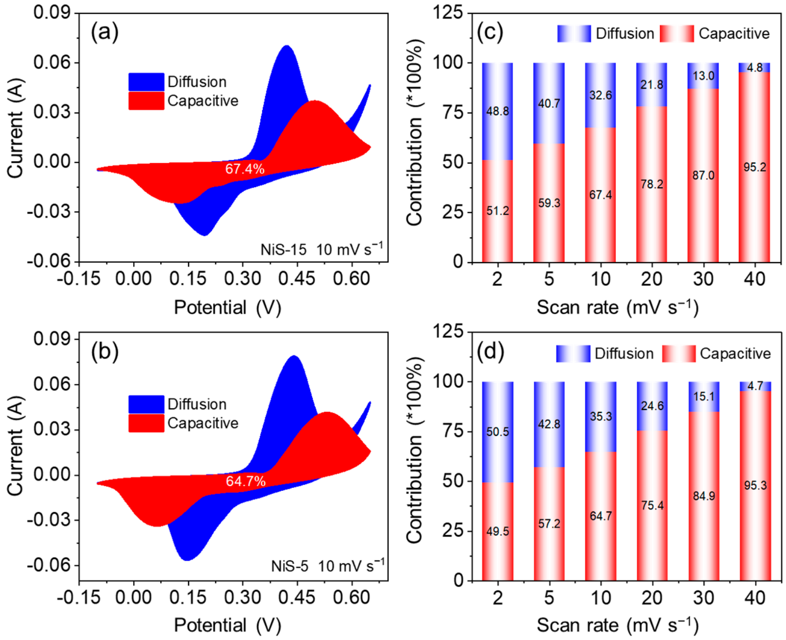

Figure S4. The contribution of surface capacitance (red) and diffusion process (blue) to the total charge storage at 10 mV s^{-1} for (a) NiS-15 and (b) NiS-5, and the contribution of surface capacitance (red) and diffusion process (blue) to the total charge storage at different scan rates for (c) NiS-15 and (d) NiS-5.

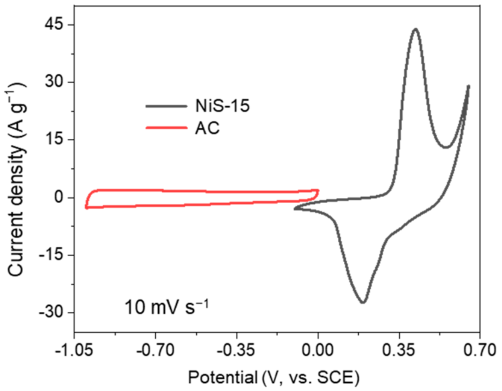

Figure S5. The CV curves of AC and NiS-15 at the scan rate of 10 mV s^{-1}.

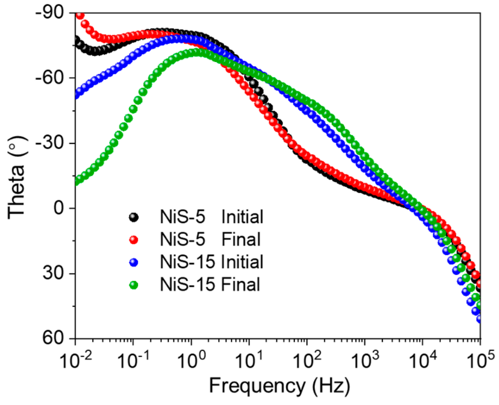

Figure S6. The Bode plots of NiS-5 and NiS-15 electrode materials.

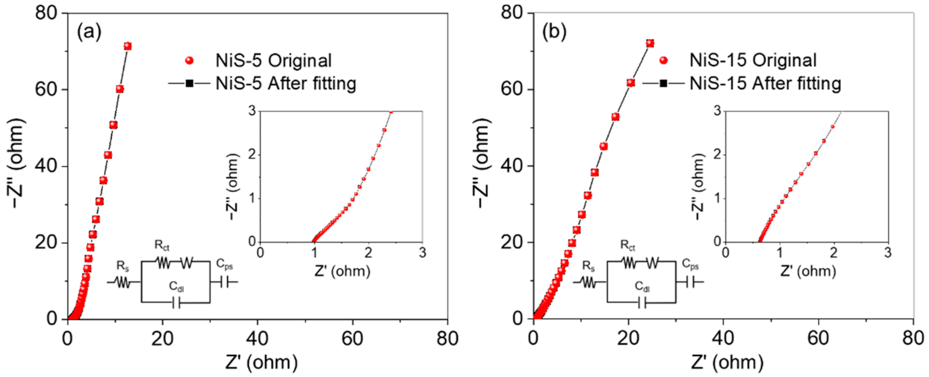

Figure S7. The Nyquist plots of (a) NiS-5 and (b) NiS-15 along with their fitting plots by ZSimDemo after cycling, and the enlarged Nyquist plots along with the corresponding simulated equivalent circuits in the insets.

Table S1. The components of the EIS equivalent circuits and their statistical parameters of modeling.

**Electrodes**	**Internal resistance (R_s/Ω)**	**Charge transfer resistance (R_{ct}/Ω)**	**Warburg impedance (Z_w/Ω $s^{-0.5}$)**	**Double layer capacitance (C_{dl}/F)**	**Pseudocapacitance (C_p/F)**
NiS-5 Initial	0.8892±0.0216	0.2544±0.0897	0.07508±0.00403	0.000337±0.000131	0.00756±0.00012
NiS-15 Initial	0.5383±0.0059	0.1000±0.0001	0.02902±0.00275	0.065460±0.000001	0.00522±0.00021
NiS-5 Final	0.9892±0.0204	1.0380±0.3793	0.05690±0.00190	0.004273±0.001302	0.00798±0.00011
NiS-15 Final	0.6953±0.0323	1.0000±0.0957	0.01692±0.00073	0.035820±0.030017	0.00365±0.00012

References

[1] Y. Wang, X. Ma, S. Li, J. Sun, Y. Zhang, H. Chen, C. Xu, Facile solvothermal synthesis of novel $MgCo_2O_4$ twinned-hemispheres for high performance asymmetric supercapacitors, J. Alloys Compd. 818 (2020) 152905.
[2] D.S. Liu, Y.F. Liu, X.H. Liu, C.J. Xu, J. Zhu, H.Y. Chen, Growth of uniform $CuCo_2O_4$ porous nanosheets and nanowires for high-performance hybrid supercapacitors, J. Energy Storage 52 (2022) 105048.
[3] H. Sun, Y. Miao, G. Wang, X. Ren, E. Bao, X. Han, Y. Wang, X. Ma, C. Xu, H. Chen, Flower-like $ZnCo_2O_4$ microstructures with large specific surface area serve as battery-type cathode for high-performance supercapacitors, J. Energy Storage 72 (2023) 108502.
[4] H. Sun, Y. Miao, G. Wang, X. Han, Y. Wang, Z. Zhang, C. Luo, X. Liu, C. Xu, H. Chen, Sonochemical synthesis of battery-type $ZnCo_2O_4$ electrode material with huge specific surface area for advanced hybrid supercapacitors, J. Energy Storage 76 (2024) 109780.
